# Prostate cancer solitary metastasis to anal canal: case report and review of literature

**DOI:** 10.1186/s12885-019-5573-9

**Published:** 2019-04-23

**Authors:** Audrius Dulskas, Vaidas Cereska, Edvardas Zurauskas, Eugenijus Stratilatovas, Feliksas Jankevicius

**Affiliations:** 1 Department of General and Abdominal Surgery and Oncology, National Cancer Institute, 1 Santariskiu Str., LT-08406 Vilnius, Lithuania; 2 Department of General and Abdominal Surgery and Oncology, National Cancer Institute, Clinic of Internal, Family Medicine and Oncology, Faculty of Medicine, 1 Santariskiu Str., LT-08660 Vilnius, Lithuania; 3 University of Applied Sciences, Faculty of Health Care, 45 Didlaukio Str., Vilnius, Lithuania; 4 National Center of Pathology, Affiliate of Vilnius University Hospital Santaros Klinikos, 5 P. Baublio Str., LT-08406 Vilnius, Lithuania; 5 Department of Urology, National Cancer Institute, 1 Santariskiu Str., LT-08406 Vilnius, Lithuania

**Keywords:** Prostate cancer, Prostate cancer metastasis, Metastatic anal canal tumour, Abdominoperineal excision, Solitary metastasis, Case report

## Abstract

**Background:**

Here we present the first cases of prostate cancer solitary metastasis to anal canal.

**Case presentation:**

A 67-year-old male patient underwent radical prostatectomy with ilio-obturator lymphonodectomy in 2016 due to poorly differentiated ductal adenocarcinoma (Gleason 4 + 5(40%) = 9) pT3bN0. Two months later increasing PSA rate was noted and the patient started adjuvant intermittent androgen deprivation therapy combined with radiotherapy. Year after patient was admitted to the hospital complaining of dyschezia, pain in anal canal, and bloody stool. Digital rectal examination revealed an anal fissure with ulceration. A biopsy from ulcerated area showed poorly differentiated ductal adenocarcinoma of the prostate. Because there was no evidence of distant metastases on abdominal computed tomography (CT) scan and pelvic magnetic nuclear resonance imaging (MRI) and the only metastasis was in anal canal patient underwent laparoscopic abdominoperineal resection (APR). Postoperative course was uneventful and patient was discharged at postoperative day 7.

**Conclusions:**

Our presented case is the first to describe prostate cancer solitary metastasis to anal canal and we always have to be aware of possible rare disease while assessing the patient with rectal bleeding. Biopsy most of the time is the only and the most reliable test to differentiate between the diseases.

## Background

Prostate cancer is a second most common cancer in males worldwide with more than 1200 million new cases diagnosed in 2018 [1]. In Lithuania it is the most common cancer in men with incidence up to 189,4 per 100,000 in 2012 [2]. The metastatic pattern of advanced prostate cancer is well known with bone tissue being the most dominant site for metastasis. Other sites of involvement are distant lymph nodes, liver, thorax, brain, digestive system, retroperitoneum, kidney and adrenal gland [3]. Finding prostate adenocarcinoma (PCa) metastasis in digestive tract is very unusual. Studies distinguishing solitary prostate cancer metastases to colon and rectum are very rare. Until now only six cases can be found in literature reporting on solitary metastasis to colon and rectum [4–9].

Here we report an exceptionally rare case of prostate adenocarcinoma solitary metastasis in anal canal. This is the first case being reported in the literature.

## Case presentation

Written informed consent was obtained from the patient for publication of this case report and accompanying images. Institutional Review Board permission was granted.

A 67-year-old male patient smoker (smokes 10 cigarettes/day) with BMI of 28.7 underwent transrectal needle biopsy of the prostate in July 2015 due to elevated prostate-specific antigen (PSA) level of 7.02 ng/mL and firm nodule with infiltration of the capsule on digital rectal examination. The patient had no previous history of faecal occult blood testing, prostate biopsies or surgeries performed. Biopsy revealed poorly differentiated ductal adenocarcinoma Gleason 4 + 4. Imunohistochemical analysis was positive for p504s and negative for PSA, CK20, CK7, and CDX2. The patient received hormonal treatment with *Leuprorelin acetate* for 9 months. Pelvic magnetic nuclear resonance imaging (MRI) showed tumour in both prostate lobes and infiltration to semi vesicles mrT3bN0 PI-RADS – 5 (Prostate Imaging Reporting and Data System). Staging of computed tomography (CT) of the abdomen and pelvis and Tc-99 m bone scan showed no radiological evidence of metastatic spread. Patient was advised to undergo surgery.

In October 2016, radical retropubic prostatectomy and ilio-obturator lymphonodectomy was performed. Pathological findings demonstrated poorly differentiated ductal adenocarcinoma (Gleason 4 + 5(40%) = 9) pT3bN0, LV1. PSA value 2 months after operation was 0,126 ng/ml and increased up to 0,358 ng/ml over four-month period which was stated as a recurrence. Patient was started on adjuvant intermittent androgen deprivation therapy combined with radiotherapy.

In August 2017, patient received 2 phase radiotherapy: first phase to prostate bed, remnant seminal vesicles and regional lymph nodes up to 50 Gy and second phase to prostate bed and remnant seminal vesicles up to 70 Gy. The PSA levels decreased, however 4 months later patient complained of mild pain in anal verge.

In May 2018, slight tenderness on 12 o’clock was noted during digital rectal examination. On rectoscopy the rectal and anal mucosa was found without any changes. Chest, abdomen and pelvic CT scan did not reveal any signs of recurrence.

**Fig. 1:**
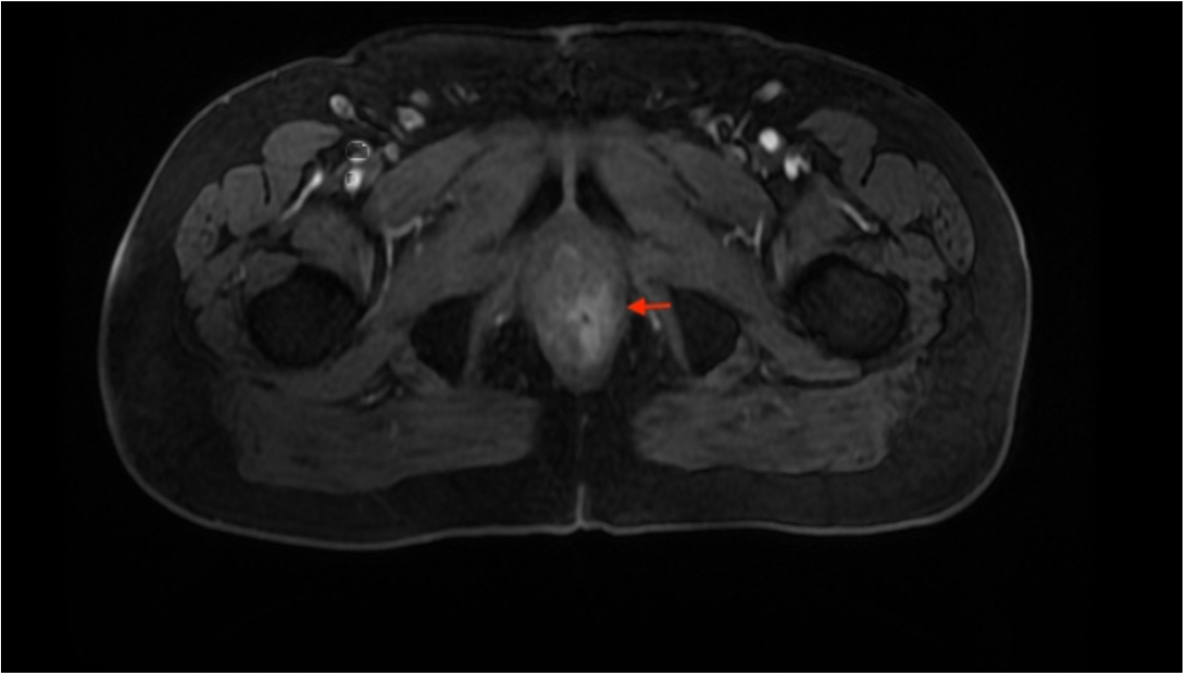
T2 transversal view. Slightly enlarged left rectum wall with a slight increase of contrast. Accumulation (red arrow)

In June 2018, the patient was admitted to the hospital complaining of dyschezia, pain in anal canal, and bloody stool. Anal inspection and palpation revealed an anal fissure with ulceration. A biopsy from ulcerated area was taken. Histologic examination reported poorly differentiated ductal adenocarcinoma of the prostate extending to muscle layer with NKX3.1, PSA, CKHMW, positive staining. MRI scan (Fig. 1) showed slightly enlarged rectum wall in anal canal with a slight increase of contrast accumulation and small nodules on the left rectum wall.

**Fig. 2:**
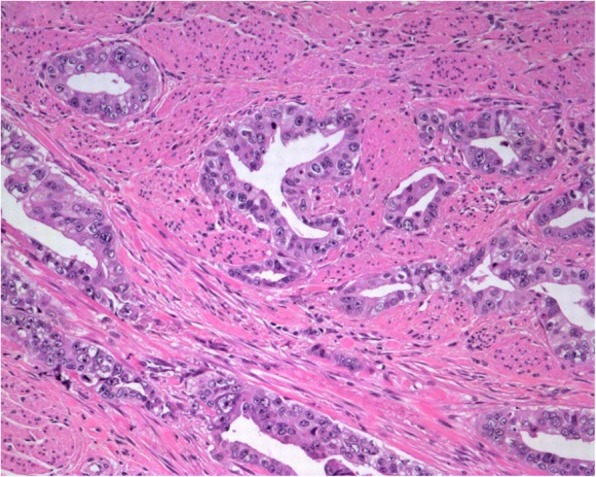
H&Ex100. Carcinoma of prostate show bowel muscular wall invasion by irregular malignant glands with enlarged nuclei, prominent nucleoli and dark cytoplasm

**Fig. 3:**
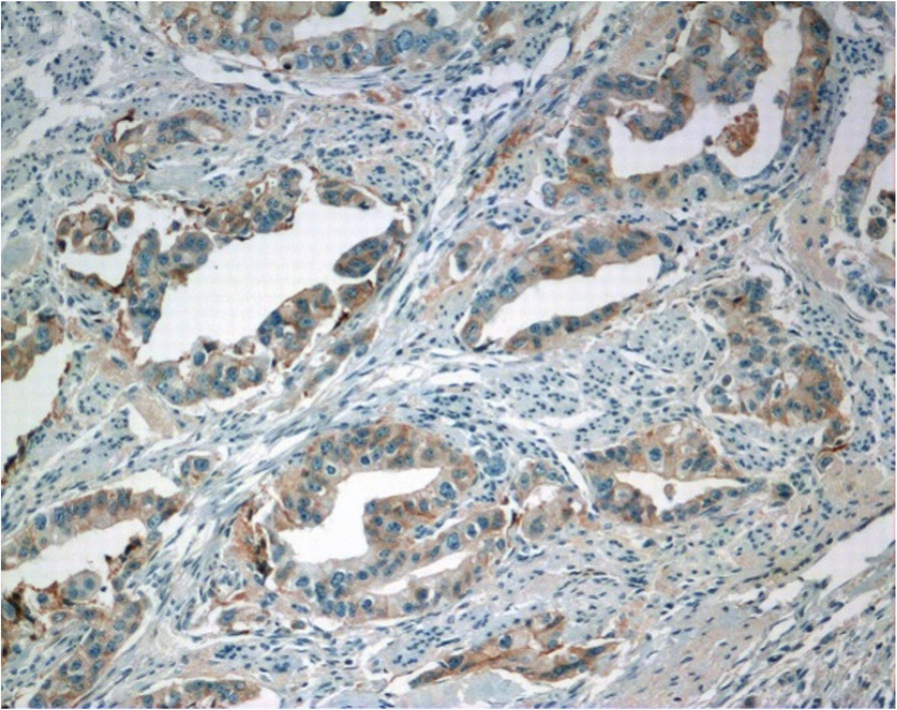
PSAx100. Malignant glands are positive for prostate specific antigen (PSA)

**Fig. 4:**
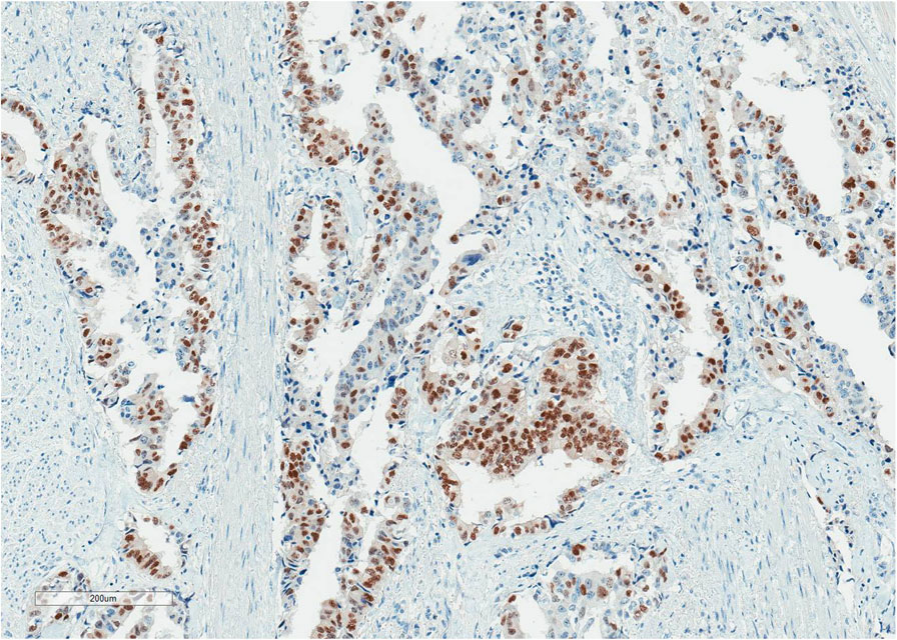
NKX3.1 × 100. Malignant glands are positive for NKX3.1

**Fig. 5:**
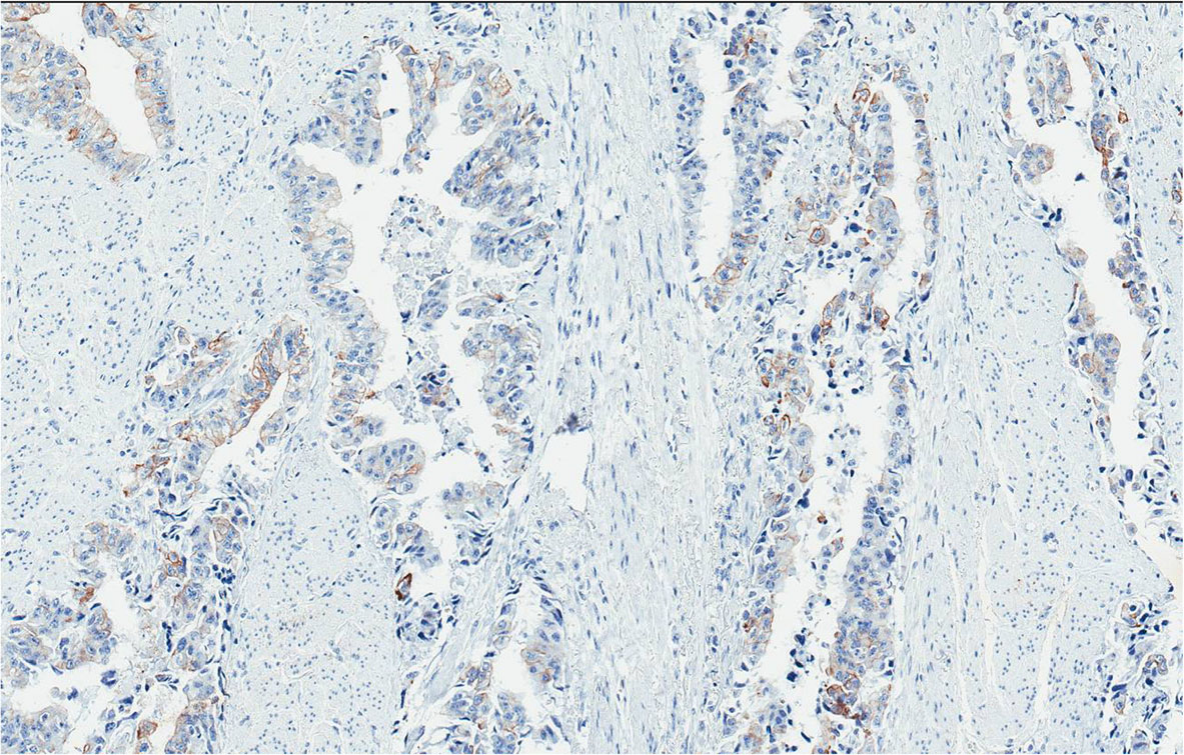
CKHMWx100. Malignant glands are positive for CKHMW

**Fig. 6:**
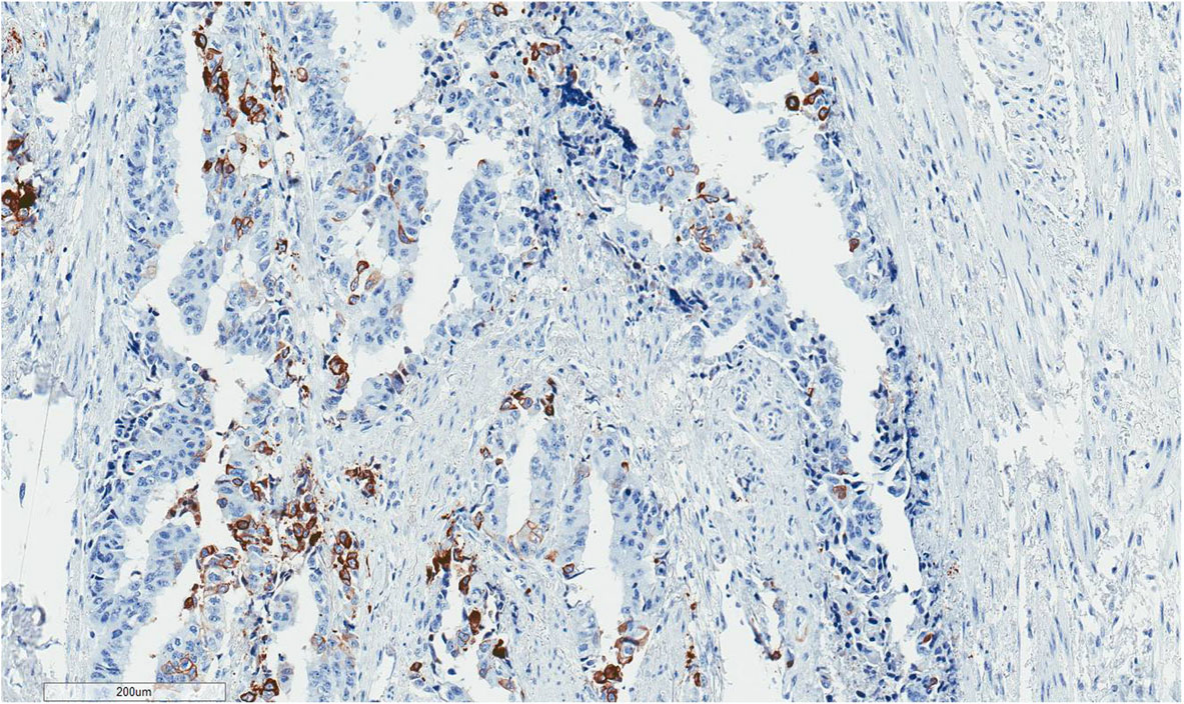
CK7x100. Malignant glands are negative for CK7

**Fig. 7:**
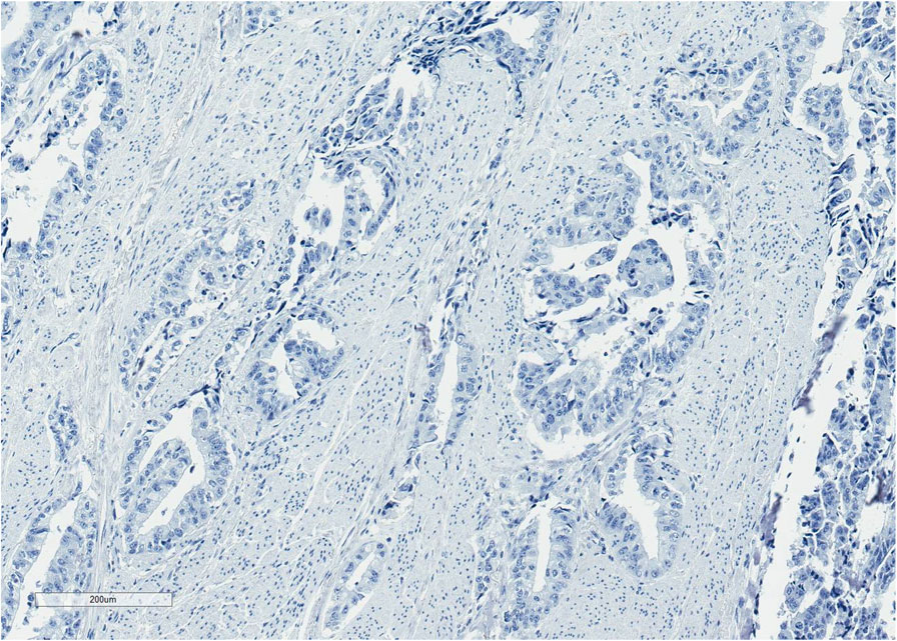
p63x100. Malignant glands are negative for p63

**Fig. 8:**
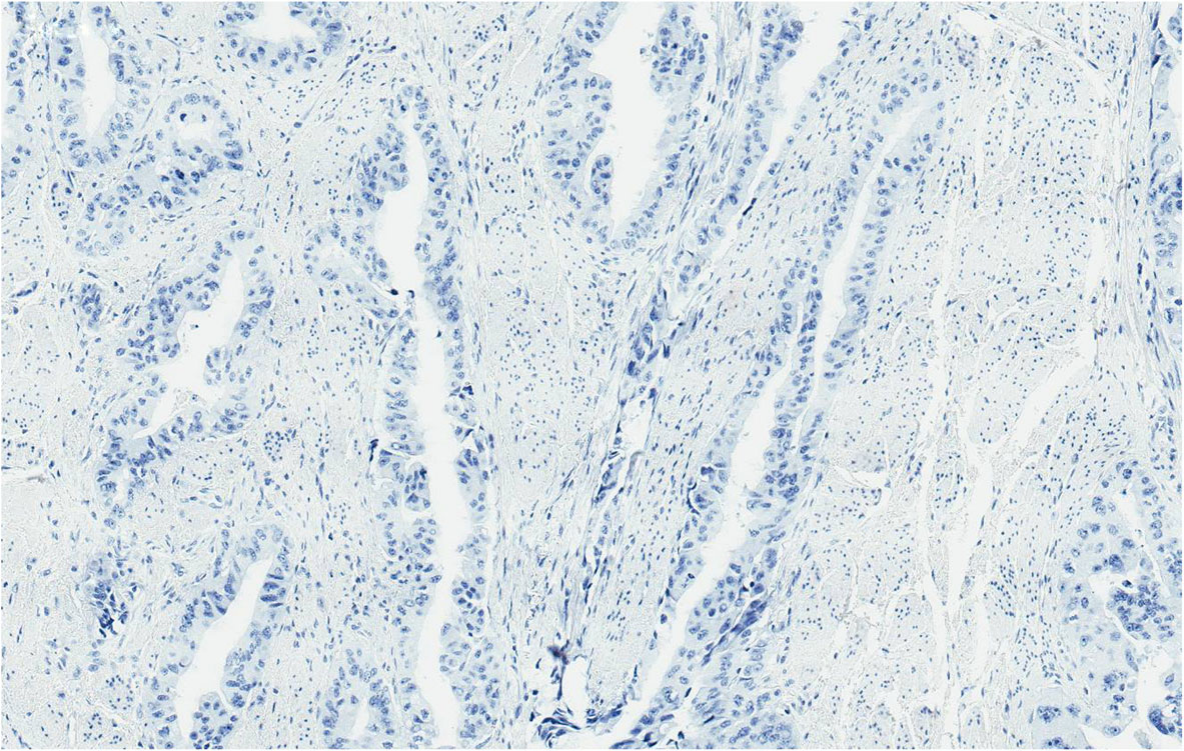
CDX2x100. Malignant glands are negative for CDX2

**Fig. 9:**
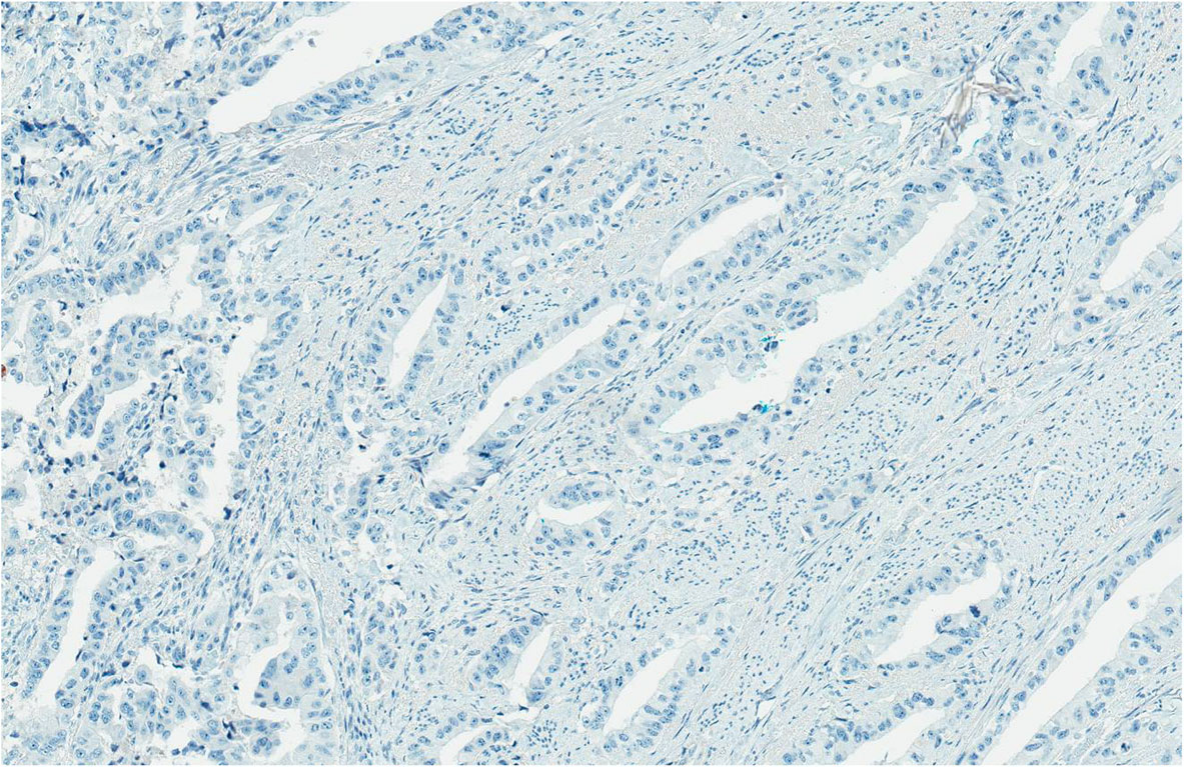
CK20x100. Malignant glands ar negative for CK20

Because of anal pain, bloody stool, prostatic metastasis verified, and no evidence of distant metastases*, the only metastasis was in the anal canal,* patient underwent laparoscopic abdominoperineal resection (APR) in August 2018. Histopathologic findings from the resected specimen showed ductal prostate cancer at the dentate line extending to the fat tissue and infiltrating mucosa, submucosa and muscle layers of the anus (Figs. 2, 3, 4, 5, 6, 7, 8 and 9). One of five lymph node had prostate cancer metastasis. Post-operative course was uneventful and on post-operative day 7, patient was discharged. Patients PSA was less than 0.2 ng/ml.

## Discussion

According to studies by *Gandaglia* et al., metastases in bone tissue account for 84–90% of advanced prostate cancer cases. Metastases in digestive system are exceptionally rare. Formerly mentioned studies report only 1, 8% and 2, 7% of patients with metastatic disease in digestive system respectively [3].

In our case solitary prostate metastasis occurred in anal canal, which is extremely rare. Previously only seven cases reporting solitary prostate metastasis to colon and rectum can be found, which are summarized in Table 1. However, radical prostatectomy, as for our patient, was only performed in two cases [4, 5]. In a case by *Vaghefi* et al. metastasis in rectum occurred 5 years after PCa diagnosis, which was treated with local excision [4]. In a second case by *Venara* et al. a prostatic carcinosarcoma occurred in rectum 10 years after initial PCa diagnosis and was treated with anterior rectum resection and radiotherapy [5]. In our case metastasis in anal canal appeared only 3 years after initial PCa diagnosis and was treated with abdominoperineal resection. Shorter period of time can be explained by relatively higher pathological Gleason sum of 9 (compared to 7 and 7 in previously mentioned cases) which has poorer prognosis.

**Table 1:** Literature review of all previous cases of solitary prostate cancer metastasis to colon and rectum

Author/Year of publication^a^	Age	Symptoms	Gleason score	Interval between original PCa diagnosis and rectal presentation	Tumour grade/Type	PSA staining in colon specimens	Treatment
Morita et al. 1991 [6]	61	obstructive defecation	n.d	5 months	G3 / adenocarcinoma	positive	AAT/total pelvic exenteration
Vaghefi et al. 2005 [4]	69	no symptoms	3 + 4 = 7	5 years	G2 / adenocarcinoma	positive	RP with pelvic lymphadenectomy/LE
Fujita et al. 2009 [7]	77	abdominal pain	n.d	same time	G3 / adenocarcinoma	positive	AAT
Venara et al. 2010 [5]	75	abdominal pain	3 + 4 = 7	10 years	prostatic carcinosarcoma	n.d	RP/ AAT and pelvic RT/intermittent hormonal treatment/ AR/radiotherapy
Nwankwo et al. 2013 [8]	69	rectal bleeding, abdominal pain	n.d	12 years	G3 / adenocarcinoma	n.d	pelvic RT/bilateral orchiectomy/ AAT/ prostate cryoablation
Liu et al. 2015 [9]	73	altered bowel habits	4 + 5 = 9	1 year	G3 / adenocarcinoma	positive	bilateral orchiectomy/AAT/ neoadjuvant chemoradiotherapy

*n.d* no data, *PSA* prostate-specific antigen, *RP* radical prostatectomy, *LE* local excision, *AR* anterior resection, *RT* radiotherapy, *APR* abdominoperineal resection, *AAT* antiandrogen therapy

^a^two cases were excluded because of the continuity invasion to colon by PCa

Symptoms of prostate cancer metastasis to rectum are similar to primary rectal cancer. Patients can complain of constipation, abdominal pain, rectal bleeding, diarrhoea, tenesmus and weight loss which can be interpreted as primary rectal cancer. In a study by *Wang* et al. almost 17% of patients with prostate cancer involving rectum were misdiagnosed as primary rectal cancer [10]. Histopathological evaluation of rectal biopsy often may be the only way to differentiate between these two diseases. Positive staining for PSA and P501s is commonly seen in prostate adenocarcinoma, unlike rectal adenocarcinoma which distinguishes with positive CDX2, CK20 and beta-catenin staining and negative for PSA and P501s [10].

In our case patient had typical rectal cancer symptoms: dyschezia, pain in anal canal, and bloody stool. Radiological findings were not specific and only histopathological examination confirmed the PCa metastasis in anal canal. As the are only few reports on rectal involvement of prostate cancer, the cancer seeding is not understood well. At least few different paths of tumour cells seeding can be identified. First direct invasion through the *Denonvilliers* fascia or through lymphatic vessels. *Vaghefi* et al. theorized the possibility of tumour cell caused by implantation in the rectum after transrectal needle biopsy [4]. Alternatively, it is possible that tumour can be spilled at prostatectomy.

## Conclusions

To conclude, our presented case is the first to describe prostate cancer solitary metastasis to anal canal and we always have to be aware of possible rare disease while assessing the patient with rectal bleeding. Biopsy most of the time is the only and the most reliable test to differentiate between the diseases.

## Abbreviations

CT scan: Computed tomography scan
LVI: Lympho-vascular invasion
MRI: Magnetic nuclear resonance imaging
PCa: Prostate adenocarcinoma
PIRADS: Prostate Imaging Reporting and Data System
PSA: Prostate specific antigen
